# Type of anesthesia and quality of recovery in male patients undergoing lumbar surgery: a randomized trial comparing propofol-remifentanil total i.v. anesthesia with sevoflurane anesthesia

**DOI:** 10.1186/s12871-021-01519-y

**Published:** 2021-12-01

**Authors:** Wenjun Meng, Chengwei Yang, Xin Wei, Sheng Wang, Fang Kang, Xiang Huang, Juan Li

**Affiliations:** Department of Anesthesiology, The First Affiliated Hospital of USTC, Division of Life Sciences and Medicine, University of Science and Technology of China, Hefei, 230036 China

**Keywords:** Anesthesia, General, Sevoflurane, Recovery, Propofol

## Abstract

**Background:**

Previous studies have shown that women achieve a better quality of postoperative recovery from total intravenous anesthesia (TIVA) than from inhalation anesthesia, but the effect of anesthesia type on recovery in male patients is unclear. This study therefore compared patient recovery between males undergoing lumbar surgery who received TIVA and those who received sevoflurane anesthesia.

**Methods:**

Eighty male patients undergoing elective one- or two-level primary transforaminal lumbar interbody fusion (TLIF) were randomly divided into two groups: the TIVA group (maintenance was achieved with propofol and remifentanil) or sevoflurane group (SEVO group: maintenance was achieved with sevoflurane and remifentanil). The quality of recovery-40 questionnaire (QoR-40) was administered before surgery and on postoperative days 1 and 2 (POD1 and POD2). Pain scores, postoperative nausea and vomiting, postoperative hospital stay, anesthesia consumption, and adverse effects were recorded.

**Results:**

The QoR-40 scores were similar on the three points (Preoperative, POD1 and POD2). Pain scores were significantly lower in the SEVO group than in the TIVA group on POD1 (30.6 vs 31.4; *P* = 0.01) and POD2 (32 vs 33; *P* = 0.002). There was no significant difference in the postoperative hospital stay or complications in the postanesthesia care unit between the TIVA group and the SEVO group.

**Conclusions:**

This study demonstrates that the quality of recovery is not significantly different between male TLIF surgery patients who receive TIVA and those who receive sevoflurane anesthesia. Patients in the TIVA group had better postoperative analgesic effect on POD2.

**Trial registration:**

This was registered at http://www.chictr.org.cn (registration number ChiCTR-IOR-16007987, registration date: 24/02/2016).

**Supplementary Information:**

The online version contains supplementary material available at 10.1186/s12871-021-01519-y.

## Background

Previously, from the perspective of doctors, desirable recovery was the rapid recovery of consciousness with stable vital signs and early discharge without complications. Currently, with increasing requirements pertaining to patient satisfaction levels and the increasing number of lumbar surgeries, anesthesiologists must consider providing fast and high-quality recovery techniques that minimize both postoperative complications and treatment stay.

A large number of studies suggest that the type of anesthesia is an important factor influencing postoperative quality of life, mostly manifesting as various discomforts, including nausea, vomiting, pain and shivering, which reduce a patient’s overall satisfaction and prolongs the length of hospital stay [1–3]. Inhalation anesthesia and total intravenous anesthesia (TIVA) are the most common general anesthesia techniques, and they have various effects on postoperative patient recovery [1, 4]. Many studies have shown that compared with desflurane anesthesia, females undergoing thyroid surgery have a significantly improved quality of recovery with TIVA. However, patient sex is an independent factor influencing postoperative recovery quality. The difference in male recovery outcomes after the administration of TIVA and volatile anesthetics remains unclear.

To meet the growing patient demand, a number of patient-centred measurement tools have been developed as a means of assessing postoperative quality of recovery [5, 6]. The Quality of Recovery-40 questionnaire (QoR-40) is one of the common methods, and it includes five dimensions with a total of 40 self-administered questions: physical comfort, physical independence, pain, emotional state, and psychological support. Previous studies have proved the validity and reliability of the questionnaire [7–10], which is suitable for Chinese people and spinal surgery [11–13].

In this study, we compared the quality of recovery between male patients undergoing lumbar surgery who received propofol and those who received sevoflurane supplemented with remifentanil. The QoR-40 was administered before surgery and 1 and 2 days post-surgery (POD1 and POD2, respectively) in male patients scheduled for transforaminal lumbar interbody fusion (TLIF) who were randomly assigned to receive either total i.v. anesthesia (TIVA group) or inhalation anesthesia (SEVO group).

## Methods

### Study design and subjects

This study method is based on Lee’s research [1]. This double-blind, randomized trial was approved by the Clinical Research Ethics Committee of The First Affiliated Hospital of USTC and was registered at http://www.chictr.org.cn (ChiCTR-IOR-16007987, Principal investigator: Chengwei Yang, registration date: 24/02/2016). Transforaminal lumbar interbody fusion (TLIF) is a common surgical method for lumbar disc herniation, using unilateral transforaminal approach, unilateral facet resection, and placement of an interbody fusion cage. Written informed consent was obtained from 80 patients undergoing elective one-level or two-level primary TLIF from 2018 to 2020 who had a primary diagnosis of spondylolisthesis, lumbar spinal stenosis, severe degenerative disc disease or facet arthropathy. The inclusion criteria were as follows: (1) males, (2) 18–64 years old, (3) body mass index (BMI) 18.5 ~ 24.9 kg/m^2^, and (4) American Society of Anesthesiologists (ASA) physical status I or II. The exclusion criteria were as follows: (1) liver and kidney dysfunction, (2) a history of central nervous system diseases, (3) language barriers or illiteracy, (4) the use of hormones, opioids, sedatives or antiemetic drugs 2 days before surgery, (5) refusal to participate in the study at any stage.

### Perioperative management

The eligible patients were randomly assigned into two equal groups (SEVO and TIVA groups) using a random-permuted block randomization algorithm via a web-based response system (www.randomization.com). Blinding was performed using opaque envelopes with number. Each envelope contain a patient’s study protocol. The researchers opened sealed envelopes before anesthesia induction. The preoperative evaluators, follow-up assessors and statisticians were blinded to the group allocation.

All subjects fasted routinely before surgery and received no premedication. Standard monitoring was conducted, which included electrocardiography, arterial blood pressure monitoring, pulse oximetry, airway pressure monitoring, capnography, and evaluation with the bispectral index (BIS VISTATM monitor, Aspect Medical Systems, Norwood, MA). In both groups, general anesthesia was induced using 1.5–2.5 mg kg^− 1^ propofol, 0.4 μg kg^− 1^ sufentanil, and 0.6 mg kg^− 1^ rocuronium. Tracheal intubation was performed in all patients using a 7.5 mm (internal diameter) tracheal tube. Mechanical ventilation was maintained with a tidal volume of 8–10 ml kg^− 1^, and partial pressure of end-tidal carbon dioxide (P_Et_CO_2_) was maintained at 35 to 45 mmHg. The carrier gas flow for both groups consisted of a combination of oxygen and air to a total flow rate of 2 L/min (fraction of inspired oxygen 0.5). Maintenance was achieved with TCI (CP-730TCI; Inc., Beijing SLGO, China) propofol (Marsh pharmacokinetic model), 1.5–3 μg ml^− 1^ propofol in the TIVA group, and sevoflurane (1.5–3.0%) in the SEVO group. For patients in both groups, analgesia was provided with remifentanil (Minto pharmacokinetic model) and sufentanil, and tropisetron hydrochloride was used as an antiemetic. Neuromuscular blockade was determined by a TOF monitor (Veryark-TOF, Guangxi, China). Rocuronium (0.15 mg/kg) was administered intravenously when T_1_/Tc values height reached 25%. BIS values were maintained ranging from 40 to 60 to monitor the depth of anesthesia. The mean arterial pressure (MAP) was maintained within 20% of the baseline value [14]. 5 min before suture, 20 ml 0.5% ropivacaine was injected into skin and subcutaneous tissues for postoperative analgesia (i.e.,10 ml per side of the incision line).

Quality of recovery was assessed before surgery and on POD1 and POD2 using the QoR-40, which included five dimensions (physical comfort, emotional state, physical independence, psychological support, and pain). The total QoR-40 score ranges from 40 (poorest quality of recovery) to 200 (best quality of recovery).

When the wound was closed, general anesthesia management for all patients was terminated, and the wake time from anesthesia began. Pain and postoperative nausea and vomiting (PONV) were measured using an 11-point numeric rating score in the postanesthesia care unit (PACU). If the score of each item exceeded 4, flurbiprofen axetil or tropisetron hydrochloride was given in PACU or ward .

In addition, the following data were also collected: perioperative MAP and heart rate (HR), consumption of remifentanil, response time (between the cessation of anesthetic maintenance drugs and the patient’s response to a verbal command), extubation time, the incidence of PONV, PACU and the postoperative hospital stay time.

### Statistical analyses

Postoperative QoR-40 score was the primary outcome of this investigation. The calculation of sample size was based on Lee’s research and our pilot study. The mean QoR-40 score of TIVA group was 174 in Lee’s research [1], and the standard deviation (SD) was 14. Based on the assumption that a 10-point difference represents a 15% improvement in the quality of recovery [13], 31 subjects per group were required to achieve a power of 80% with a type 1 error of 0.05. Considering a 20% drop-out rate, 80 subjects were enrolled.

SPSS version 16.0 software (SPSS Inc., Chicago, IL) was used for statistical analysis. Continuous variables are expressed as mean ± standard deviation or median (interquartile range). If the data meet the normality, the t-test was used for inter group comparison. Otherwise, the non-parametric test was used for inter group comparison. A *P*-value of < 0.05 was considered statistically significant.

## Results

**Fig. 1:**
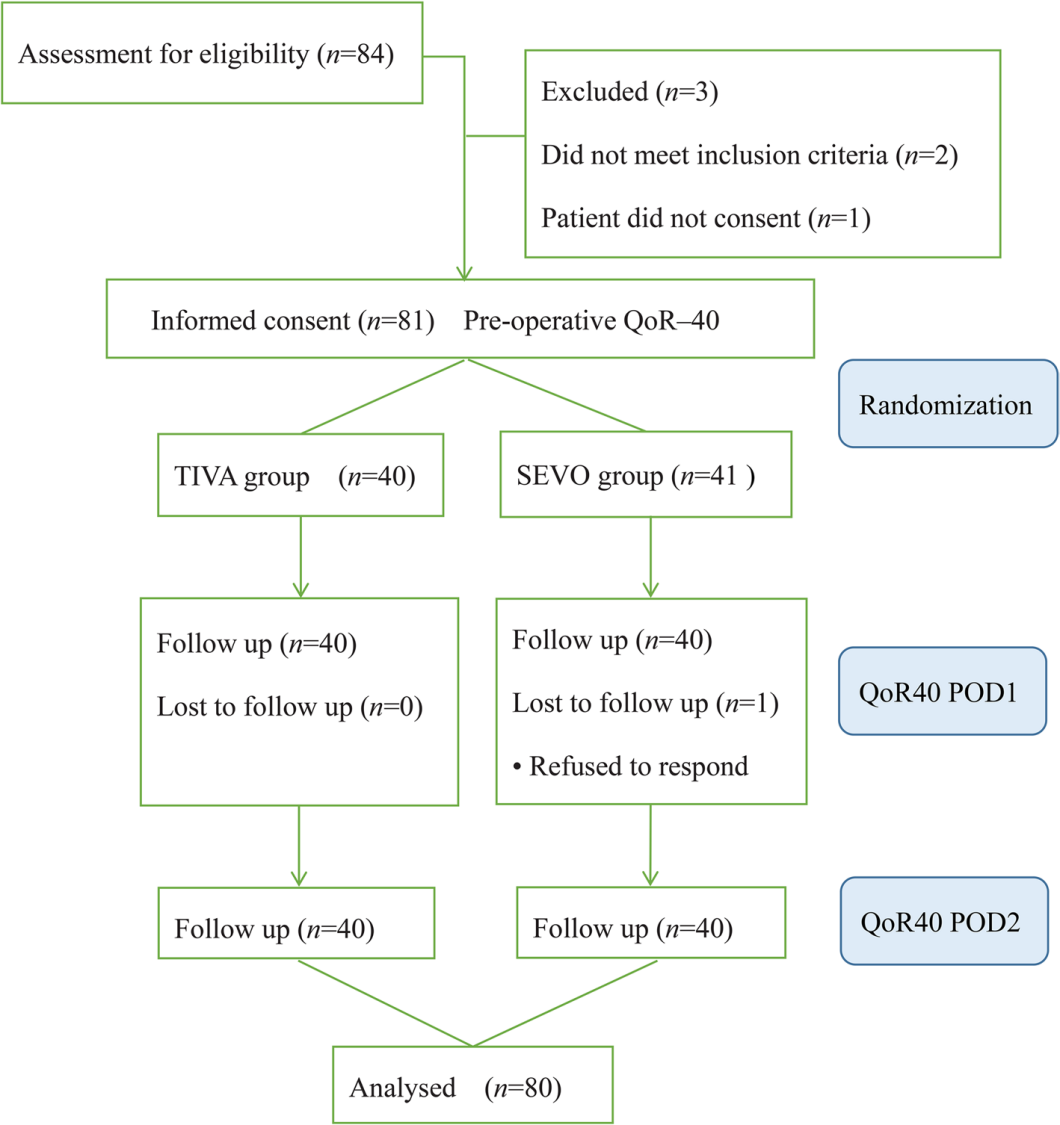
A flowchart that outlines patient selection, randomization,and analysis

Among the 84 patients who underwent TLIF, 80 patients met our inclusion criteria and were randomly assigned to the study groups. After excluding 4 patients for different reasons, data analysis was performed on the 80 patients. The flowchart in Fig. 1 shows the number of patients at each stage of the study. The study population characteristics are presented in Table 1. There was no significant difference between the groups in terms of age, BMI, anesthetic duration, operation time, or postoperative hospital stay.

**Table 1:** Patient characteristics of patients in the TIVA and SEVO groups

	SEVO group(*n* = 40)	TIVA group(*n* = 40)
Age, mean (SD), (yr)	50.9 (8.9)	48.8 (8.1)
Height, mean (SD), (m)	1.73 (6.48)	1.73 (4.88)
Weight, mean (SD),(kg)	68.71 (6.82)	68.66 (6.12)
BMI,^a^ mean (SD)	23.0 (1.4)	23.0 (1.5)
ASA physical status I/II	5/35	3/37
Preoperative comorbidities		
Hypertension	6 (15%)	7 (17.5%)
Diabetes mellitus	1 (2.5%)	2 (5%)
Old cerebral infarction	1 (2.5%)	1 (2.5%)
Operative segment single/double	17/23	15/25

*IQR* Inter-quartile range, *SD* Standard deviation. ^a^Calculated as kg m^− 2^

The preoperative, POD1, and POD2 QoR-40 scores are presented in Table 2. The QoR-40 scores were similar on the three points. Pain scores were significantly lower in the SEVO group than in the TIVA group on POD1 (30.6 vs 31.4; *P* = 0.01) and POD2 (32 vs 33; *P* = 0.002). Regarding the scores on all dimensions, the most obvious change was a significantly reduced number of physical independence points on POD1 than preoperatively, however, these scores improved on POD2.

**Table 2:** Effectiveness outcomes. QoR-40, quality of recovery-40 questionnaire

	SEVO group (*n* = 40)	TIVA group (*n* = 40)	*P*-value	Difference (95% CI)
**Preoperative**				
Emotional status, mean (SD)	39.7 (2.2)	39.9 (2.3)	0.764	−0.15(−1.14 to 0.84)
Physical comfort, mean (SD)	54.0 (2.8)	55.3 (2.9)	0.051	−1.25(−2.51 to 0.01)
Psychological support, median (IQR)	33 (32–34)	33 (32–34)	0.916	–
Physical independence, median (IQR)	23 (22–24)	23 (22–24)	0.689	–
Pain, mean (SD)	30.2 (2.0)	31.0 (1.6)	0.055	−0.80(−1.62 to 0.02)
Total QoR-40, mean (SD)	179.6 (5.4)	181.7 (5.6)	0.089	−2.13(−4.58 to 0.33)
**POD1**				
Emotional status, mean (SD)	40.1 (2.1)	39.9 (2.4)	0.691	0.20(−0.80 to 1.20)
Physical comfort, mean (SD)	54.2 (2.4)	53.5 (2.9)	0.226	0.73(−0.46 to 1.91)
Psychological support, median (IQR)	33 (32–33)	33 (32–33)	0.667	–
Physical independence, mean (SD)	15.4 (1.9)	16.0 (2.5)	0.171	−0.68(−1.65 to 0.30)
Pain, mean (SD)	30.6 (1.2)	31.4 (1.3)	0.010	−0.75(−1.32 to − 0.18)
Total QoR-40, mean (SD)	173.0 (5.4)	174.5 (5.4)	0.681	−0.5(−2.91 to 1.91)
**POD2**				
Emotional status, median (IQR)	42 (40–42)	41 (40–42)	0.338	–
Physical comfort, mean (SD)	55.7 (2.1)	55.4 (3.2)	0.59	0.33(−0.87 to 1.52)
Psychological support, median (IQR)	33 ((33–33)	33 (32–34)	0.963	–
Physical independence, mean (SD)	16.8 (2.4)	17.2 (2.2)	0.505	−0.35(−1.39 to 1.69)
Pain, median (IQR)	32 (31–33)	33 (32–33)	0.002	–
Total QoR-40, mean (SD)	178.5 (5.1)	178.8 (5.5)	0.818	−0.28(−2.64 to 2.09)

*POD* Postoperative days. *TIVA* Total i.v. anesthesia. SEVO, sevoflurane. *SD*, standard deviation; *IQR*, inter-quartile range

**Fig. 2:**
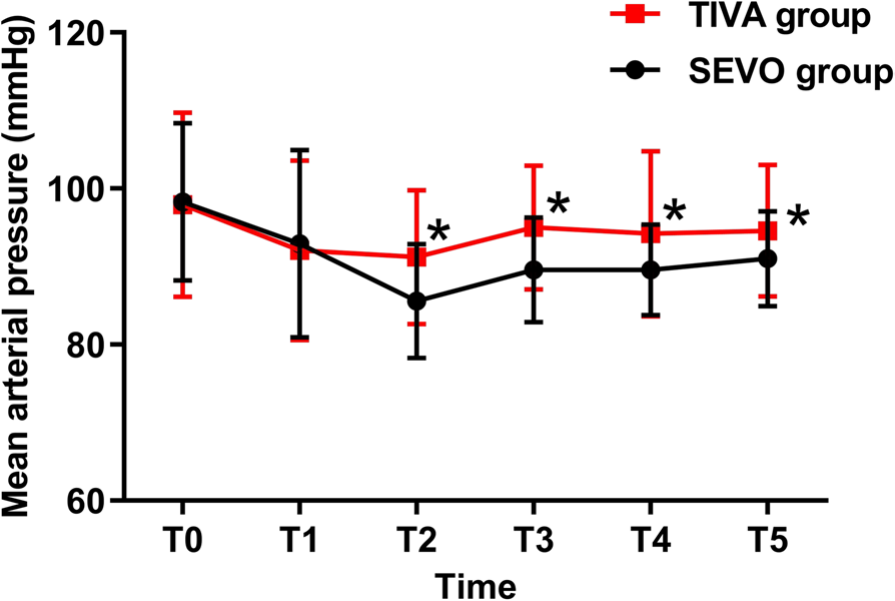
Perioperative MAP comparisons between the TIVA and SEVO groups. MAP, mean arterial pressure; T0, preoperative; T1, 10 min after induction; T2, cessation of main anesthetics; T3, tracheal extubation; T4, admission to PACU; T5, discharge from PACU

The perioperative data are showed in Table 3. There was no significant difference in the hospital stay or complications in the PACU between the TIVA group and the SEVO group. MAP was significantly higher in the TIVA group upon cessation of main anesthetics (85.6 vs 91.2; *P* = 0.002), tracheal extubation (89.6 vs 95.0; *P* = 0.001), entering the PACU (89.6 vs 94.2; *P* = 0.018) and leaving the PACU (91.0 vs 94.6; *P* = 0.028). (Fig. 2).

**Table 3:** Perioperative variables

	SEVO group (*n* = 40)	TIVA group (*n* = 40)	*P*-value
Anesthetic duration,mean (SD) (min)	129.2 (34.7)	129.5 (33.2)	0.969
Operation time, mean (SD) (min)	108.0 (30.1)	106.9 (29.6)	0.872
Transfusion volume,mean (SD) (ml)	1420 (429)	1428 (314)	0.29
Blood loss, median (IQR) (ml)	100 (100–150)	100 (50–100)	0.17
Remifentanil usage, mean (SD) (ug)	813 (223)	838 (272)	0.662
Time to obeying commands, median (IQR) (min)	8.2 (7.7–10.3)	7.9 (6.8–10.2)	0.089
Tracheal extubation,median (IQR)(min)	9.5 (7.9–12.5)	10.1 (8.8–11.6)	0.242
PACU			
Duration in PACU, mean (SD) (min)	43.6 (6.9)	45.2 (6.9)	0.311
Vomiting and Nausea	3 (7.5%)	1 (2.5%)	0.305
Pain	1 (2.5%)	2 (5%)	0.556
Agitation	0	1 (2.5%)	0.314
VAS score			
preoperative, mean (SD) (min)	4.40 (1.68)	4.35 (1.78)	0.897
PACU,median (IQR)	2 (2–2)	2 (2–2)	0.198
POD1, median (IQR)	3 (2–3.75)	3 (2–3)	0.347
POD2,median (IQR)	2 (2–3)	2 (2–2))	0.001
Postoperative hospital stay, median (IQR) (days)	4 (3–5)	4 (3–5)	0.658
Postoperative analgesia			
Day1 (%)	10 (25%)	9 (22.5%)	0.793
Day 2 (%)	4 (10%)	7 (17.5)	0.456

*IQR* Inter-quartile range, *SD* Standard deviation. t0, preoperative

**Fig. 3:**
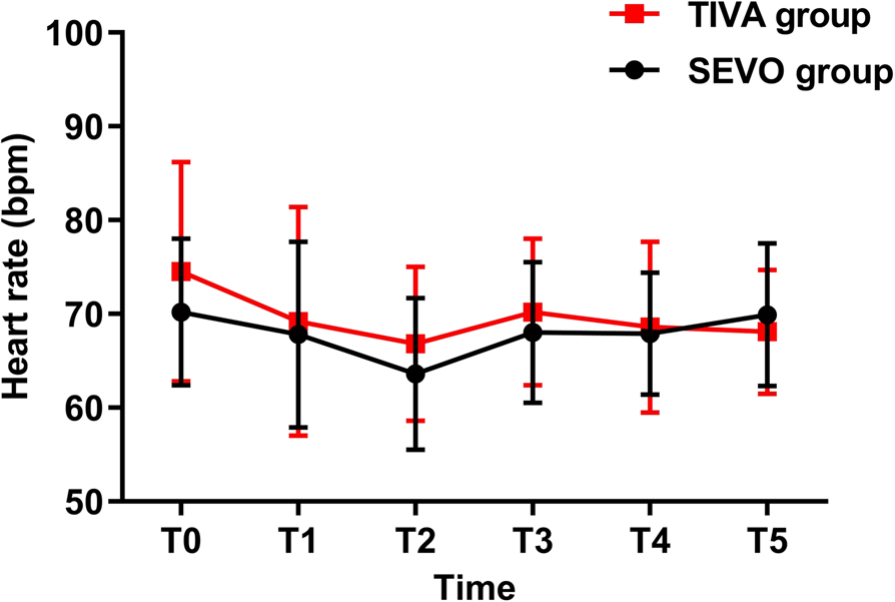
Perioperative HR comparisons between the TIVA and SEVO groups. HR, heart rate; T0, preoperative; T1, 10 min after induction T2, cessation of main anesthetics; T3, tracheal extubation; T4, admission to PACU; T5, discharge from PACU

There was no significant difference in heart rate between two groups (Fig. 3). The obeying command time and tracheal extubation time were similar between the two groups. During the PACU stay, three patients in the SEVO group and one patient in the TIVA group complained of PONV and did not use additional antiemetics. One patient in the SEVO group and two patients in the TIVA group experienced pain, and one patient in the TIVA group received pain relief treatment in the PACU. Although the amount of intraoperative remifentanil administered was higher in the TIVA group, this difference was not significantly different between the two groups (813 vs 838; *P* = 0.662). VAS score was significantly higher in the SEVO group upon POD2. The use of postoperative analgesics was similar between the two groups in the ward.

The date are showed in Tables 4 and 5, which comparing the QoR-40 scores between three measure points in the TIVA group or SEVO group. Total QoR-40 scores were significantly lower in the two groups on POD1. Compared preoperative, physical independence scores were significantly lower in the two groups on POD1 and POD2.

**Table 4:** Compare the QoR-40 scores (global and sub-dimensions) between three measure points in the TIVA group

	Emotional status	Physical comfort	Psychological support	Physical independence	Pain	Total QoR-40
Preoperative	39.9 (2.3)	55.3 (2.9)	33 (32–34)	22.9 (1.3)	31.0 (1.6)	181.7 ± 5.60
POD1	39.9 (2.4)	53.5 (2.9)	33 (32–33)	16.0 (2.5)	31.4 (1.3)	173.5 ± 5.40
p	0.88	0.001	0.396	0.00	0.227	0.00
CI	−0.05 (−0.70 to − 0.60)	1.8 (0.74 to 2.85)	–	6.9 (6.06 to 7.68)	−0.35 (− 0.93 to 0.23)	8.17 (6.58 to 9.77)
Preoperative	39.9 (2.3)	55.3 (2.9)	33 (32–34)	22.9 (1.3)	31.0 (1.6)	181.7 ± 5.60
POD2	40.7 (2.0)	55.4 (3.2)	33 (32–34)	17.2 (2.2)	32.6 (1.0)	178.8 ± 5.51
p	0.018	0.877	0.034	0.00	0.00	0.002
CI	−0.85 (−1.55 to − 1.53)	− 0.075 (− 1.05 to 0.90)	–	5.75 (5.01 to 6.49)	− 1.6 (−2.1 to − 1.1)	2.9 (1.13 to 4.72)

*QoR-40* Quality of recovery-40 questionnaire. *POD* Postoperative days. *TIVA* Total intravenous anesthesia. *SD*, standard deviation; *IQR*, inter-quartile range

**Table 5:** Compare the QoR-40 scores (global and sub-dimensions) between three measure points in the SEVO group

	Emotional status	Physical comfort	Psychological support	Physical independence	Pain	Total QoR-40
Preoperative	40 (38–41)	54.0 (2.8)	33 (32–33)	22.9 (1.1)	30 (29–31)	179.6 (5.4)
POD1	41 (38–42)	54.2 (2.4)	33 (32–33)	15.4 (1.9)	31 (30–31)	173.0 (5.4)
p	0.099	0.664	0.863	0.00	0.05	0.00
CI	–	−0.18 (−0.98 to 0.63)	–	7.5 (6.86 to 8.14)	–	6.6 (4.90 to 8.20)
Preoperative	39.7 (2.2)	54.0 (2.8)	33 (32–33)	22.9 (1.1)	30.2 (2.0)	
POD2	40.2 (1.5)	55.7 (2.1)	33 (33–33)	16.8 (2.4)	31.8 (1.1)	178.1 (5.1)
p	0.00	0.00	0.05	0.00	0.00	0.099
CI	−1.48 (−2.01 to −0.94)	−1.65 (−2.16 to − 1.14)	–	6.05 (5.36 to 6.74)	− 1.63 (− 2.26 to-0.99)	1.07 (− 0.21 to 2.36)

*QoR-40* Quality of recovery-40 questionnaire. *POD* Postoperative days. *SEVO* Sevoflurane. *SD*, standard deviation; *IQR*, inter-quartile range

## Discussion

We found that male patients had similar QoR-40 scores on POD1 and POD2 compared with the preoperative QoR-40 scores in those receiving TIVA or sevoflurane anesthesia undergoing TLIF. Pain scores were significantly higher in the TIVA group than in the SEVO group on POD1, and this difference seemed to persist on POD2. Our results showed that the total scores of the two groups of patents decreased on POD1 compared with preoperative scores, a result that was consistent with previous studies [1, 11].

Previous studies have confirmed that that gender is an independent factor influencing postoperative recovery, and men emerged slower from general anesthesia and have better overall recovery quality [4]. while another study demonstrated that female patients have significantly better recovery quality with TIVA than with inhalation anesthesia [1]. Few studies have compared the quality of recovery between TIVA and inhalation anesthesia from the male patient perspective. Our study concluded that the anesthetic method does not influence male patient-perceived quality of recovery.

The type of anesthesia has been proved to be a factor affecting the incidence of postoperative pain [15, 16]. In our results, the most significant differences between the TIVA and SEVO groups were in the pain dimension on POD1 and POD2, and this finding is similar with the results of most previous studies [15, 17–20]. VAS score was significantly higher in the SEVO group on POD2, which was consistent with the result of QoR-40 pain score, indicating that patients in the TIVA group had better postoperative analgesic effect. Some reports have shown that propofol application can affect intrinsic analgesic effect, manifested as the decrease of postoperative analgesic consumption and the absence of hyperalgesia [21, 22]. Propofol can interact with GABA_A_ and glycine receptors, which block the nociceptive transmission of neurons and peripheral nociceptive neurons [19, 23]. In addition, high dose remifentanil can cause hyperalgesia, previous studies found that propofol not only may prevent remifentanil-induced hyperalgesia caused by high-dose remifentanil [19], but also inhibits the N-methyl-D-aspartate (NMDA) subtype of the glutamate receptor [23], which may be the reason why the TIVA group was associated with more remifentanil usage and better analgesic effects in our study.

To date, the effect of anesthesia type on PONV has been uncertain [24, 25]. Most previous studies have suggested that TIVA anesthesia with propofol for the maintenance of general anesthesia decreases the risk of PONV [26]. Additionally, volatile anesthetics have been shown to increase the risk of PONV in surgery patients [27]. However, when propofol is given as a maintenance regimen, it may have a clinically relevant effect on PONV in the short term. In the present study, although we found that the incidence of PONV in the SEVO group was higher than that in the TIVA group, the difference was not statistically significant. The low incidence of PONV in the male population may be the reason why there was no significant difference between the two groups in our study.

### Limitations

There were several limitations to this study. First, the age of the recruited patients was relatively low, with an average age under 65 years for the TIVA and SEVO groups. Therefore, the results may not be as generalizable to older patients. Second, the sample size was calculated for the detection of differences in the total QoR-40 score and may be inadequate for comparing each of the different dimensions between the groups. Third, our trial focused on patients who were healthy and male and who were undergoing elective TLIF. Thus, we cannot comment on whether the conclusion would be different in patients undergoing complex surgery or those with serious comorbidities.

## Conclusions

In conclusion, among male patients undergoing elective TLIF surgery, an intraoperative anesthetic regimen that included volatile anesthetics did not result in significant differences in postoperative quality of recovery on POD1 or POD2 compared with a regimen of total intravenous anesthesia. Patients in the TIVA group had better postoperative analgesic effect on POD2.

## Supplementary Information

**Additional file 1.**

## Data Availability

The datasets used and/or analysed during the current study are available from the corresponding author on reasonable request.

## Abbreviations

TIVA: Total intravenous anesthesia
SEVO: Sevoflurane
TLIF: Transforaminal lumbar interbody fusion
QoR-40: Quality of recovery-40 questionnaire
POD: Postoperative days
BIS: Bispectral index
P_et_CO_2_: Partial pressure of end-tidal carbon dioxide
TCI: Target controlled infusion
PONV: Postoperative nausea and vomiting
PACU: Postanesthesia care unit
NMDA: N-methyl-D-aspartate
